# Association between arthritis and hand grip strength indices combined with anthropometry in an older Korean population

**DOI:** 10.1371/journal.pone.0291046

**Published:** 2023-08-31

**Authors:** Jeong H. Chi, Bum J. Lee

**Affiliations:** 1 Department of Computer Science and Engineering, Konkuk University, Seoul, Republic of Korea; 2 Digital Health Research Division, Korea Institute of Oriental Medicine, Deajeon, Republic of Korea; Universidade Federal de Sao Carlos, BRAZIL

## Abstract

**Background:**

Hand grip strength (HGS) is related to arthritis and all-cause mortality. Many studies have examined the association between HGS and arthritis, but these studies did not consider relative HGS indices. The objectives of this study were to examine the association between arthritis and HGS indices in an older Korean population and to compare an absolute HGS index and a relative HGS indices.

**Methods:**

In a large-scale cross-sectional study, a total of 16,860 subjects older than 50 years from the Korea National Health and Nutrition Survey from 2014 to 2019 were included for statistical analysis. A binary logistic regression model was used to examine the association between arthritis and HGS indices in crude and covariate-adjusted models.

**Results:**

In the crude analysis, all anthropometric and HGS indices were associated with arthritis except for weight in men. In adjusted models 1 and 2, among the anthropometric indices, waist circumference (WC) and waist-to-height ratio (WHtR) were associated with arthritis in men but not in women. Absolute HGS and all relative HGS indices showed a negative association with arthritis among both men and women, and the magnitude of the association of arthritis with the absolute HGS index and the relative HGS indices was similar. However, the magnitude of the association between all HGS indices and arthritis was higher for men than for women except in the crude analysis.

**Discussion:**

Absolute and relative HGS indices had negative associations with arthritis, and the magnitude of the association between the absolute HGS index and arthritis and between the relative HGS indices and arthritis was similar in all models. To our knowledge, this is the first report of an association between arthritis and relative HGS indices, which was not observed in previous studies.

## Introduction

Hand grip strength (HGS), which reflects overall muscle strength, is a cost-effective, reliable, quick, and easy measure of many diseases [1, 2]. HGS is closely associated with cardiovascular mortality [1–3], all-cause mortality [4], visual impairment and diabetic retinopathy [5], alexithymia [6], myalgic encephalomyelitis/chronic fatigue syndrome [7], nonalcoholic fatty liver disease [8], osteoporosis [9], and arthritis [10–22]. Normative HGS is strongest among individuals aged 40–50 years and gradually decreases with age in both men and women [23, 24]. Additionally, HGS is closely related to sex, age, height, occupation, and weight [23, 24]. Therefore, several previous studies have reported normative or reference HGS values that consider age, height, and the dominant arm among middle-aged and older men and women [25], among young men and women [26, 27], among children and adolescents (boys and girls) [28], and in all age groups [29] in various countries.

HGS is commonly used as an outcome measure in arthritis trials. Arthritis is a chronic systemic disease related to joint information that causes disability, joint pain, stiffness, and swelling [30, 31]. In general, modifiable risk factors for arthritis are overweight and obesity, smoking, periodontitis, nutrition, and osteoarthritis [30–34]. Nonmodifiable risk factors for arthritis are aging, female sex, family history of rheumatoid arthritis, multiple genetic factors such as HLA-DRB1 alleles and peptidyl arginine deaminase-4, low education level, high birth weight, and environmental risks such as air pollution, silica dust, solvents, ultraviolet light and, in rheumatoid arthritis, hormones related to women [30–34]. As the link between grip strength and arthritis becomes more prominent, several studies have reported protocols or assessments of HGS measurements in arthritis patients [16–19].

To date, studies have been conducted to determine the association between HGS and arthritis in many countries [10–22]. These studies have reported that higher HGS contributes to a decreased prevalence of arthritis, and HGS is a useful screening tool for arthritis. However, these studies did not consider relative HGS indices; instead, only the absolute HGS index was considered to examine the association between HGS and arthritis. The objectives of this study were to examine the association between arthritis and HGS indices in an older Korean population and to compare the magnitude of the associations of the absolute and relative HGS indices suggested by this study. Our results provide fundamental guidelines for the design of screening tools for arthritis.

## Materials and methods

### Study population and data sources

**Fig 1:**
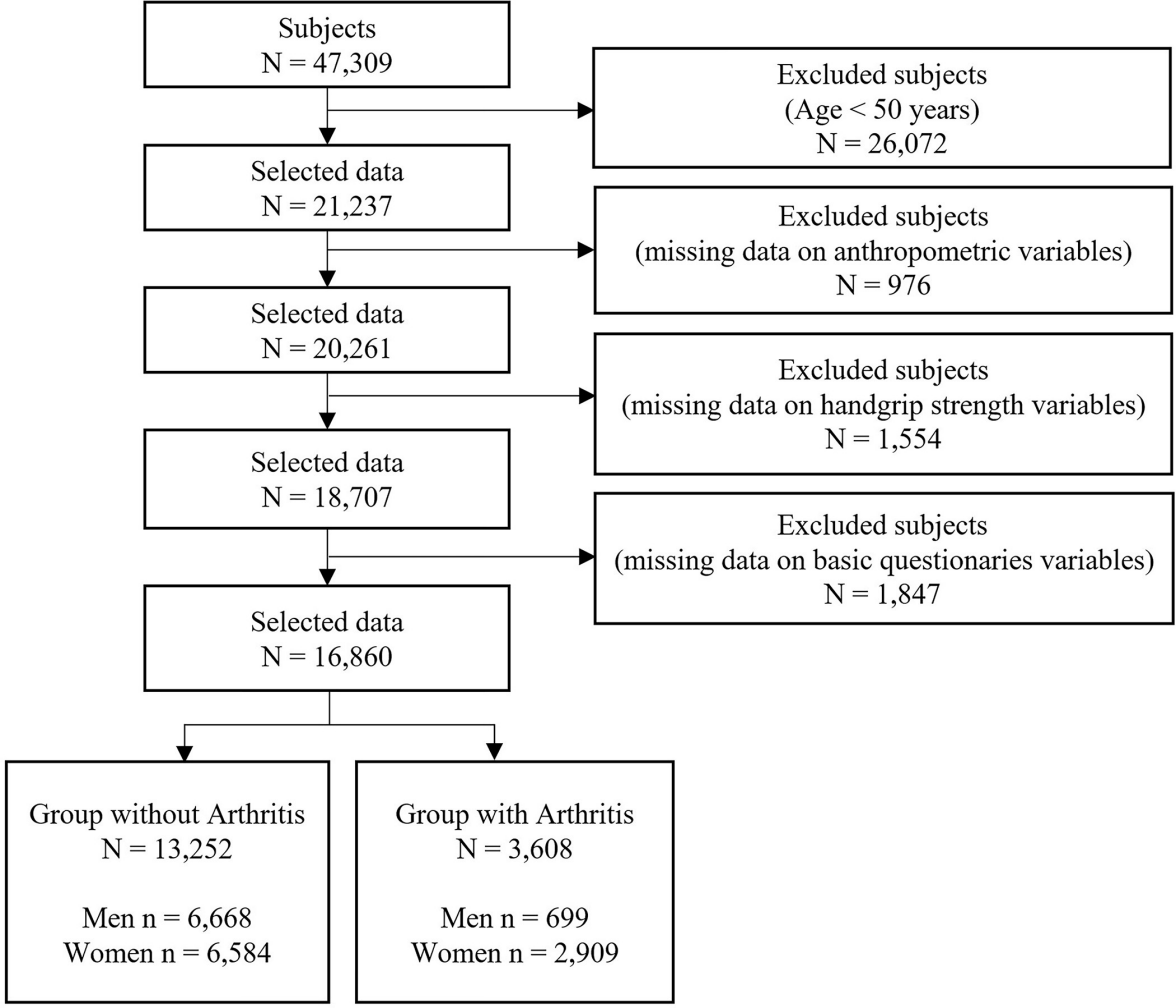
Sample selection procedure.

The Korean National Health and Nutrition Examination Survey (KNHANES) is an annual national health and nutrition survey conducted to produce nationally representative and reliable statistics on the health status, health behaviors, and food and nutrition intake of Korean people [35, 36]. From 2014 to 2019, HGS was measured to identify muscle strength distribution. In 2020, HGS measurement was stopped to prevent the spread of coronavirus disease 2019 (COVID-19). In this large-scale cross-sectional study, we used the KNHANES dataset from 2014 to 2019, which included HGS and arthritis [35]. A total of 47,309 (men = 21,566, women = 25,743) subjects participated in the health interview survey and the health examination, both of which were conducted in a mobile examination vehicle. The health survey was conducted through an interview and self-report method, and all subjects in the survey submitted written informed consent. The KNHANES datasets were approved by the Korea Centers for Disease Control and Prevention (IRB: 2010-02CON-21-C, 2011-02CON-06-C, 2012-01EXP-01-2C, 2013-07CON-03-4C, 2013-12EXP-03-5C, 2018-01-03-P-A, 2018-01-03-C-A). Additionally, this study, based on KNHANES data, received ethics approval from the Institutional Review Board of the Korea Institute of Oriental Medicine (KIOM) (IRB No. I-2209/009-001 and I-2202/002-001). This study covered adults older than 50, and 16,860 subjects were ultimately chosen based on the inclusion and exclusion criteria. Fig 1 shows the detailed sample selection procedure. This study was conducted in accordance with the Declaration of Helsinki. All methods were performed in accordance with the relevant guidelines and regulations.

### Definition of arthritis

Data on arthritis were collected through face-to-face health interviews with well-trained staff and determined by responses to two questions [35]: “Do you have osteoarthritis diagnosed by a doctor?” and “Do you have rheumatoid arthritis diagnosed by a doctor?” Subjects who answered “yes” to at least one of the two questions were placed in the arthritis group, and those who answered “no” to the questions were placed in the nonarthritis group. To overcome respondent recall bias regarding the diagnosis of arthritis, health interview surveys were performed during face-to-face health interviews conducted by experts or well-trained staff according to the guidelines for each item rather than by self-administered questionnaires [35, 36].

### Measurement

Through health interviews and a self-report method, demographic and health behavior variables such as sex, age, residential area, marital status, family type, education level, occupation, household income, stress, alcohol consumption, smoking status, walking exercise, sleep duration, and menopause were collected. In the health examination, height and weight were measured in units of 0.1 cm and 0.1 kg, respectively, using an automatic measuring device (JENIX DS-102, Dong Sahn Jenix Co., Seoul, Korea). BMI was calculated by dividing weight (kg) by height squared (m2). Waist circumference was measured in units of 0.1 cm using a tape measure (Seca 200, Hamburg, Germany). WHtR was calculated by dividing WC by height. Systolic and diastolic blood pressure were measured three times with a standard mercury sphygmomanometer (Baumanometer Wall Unit 33(0850)/USA) and then calculated as the average value of the second and third measurements [35–37]. HGS was not measured for those who experienced discomfort and had functional limitations that made it difficult to measure HGS, such as a history of wrist surgery or pain within the last 3 months. HGS was measured using a digital grip strength dynamometer (TTK 5401, Takei Scientific Instruments Co., Ltd., Japan) while the participants stood with both feet shoulder-width apart and kept their elbows or wrists out of contact with their torso. HGS was measured alternately starting with the dominant hand (right hand, left hand, both hands) and was measured three times each with a rest period of 1 minute [35]. In this study, we used the average of the three trials of the predominant hand as the absolute HGS. The values obtained by dividing the absolute HGS by body measurement information, such as height, weight, BMI, WC and WHtR, were used as the relative HGS indices.

### Statistical analysis

KNHANES aims to produce statistics that represent the Korean population by utilizing data extracted from the Population and Housing Census and the Joint Housing Price Disclosure data as the sampling frame. KNHANES employs a two-stage stratified cluster sampling method with survey areas and households as the first- and second-stage sampling units, respectively. Additionally, it selects samples based on inherent stratification criteria such as region, urban/rural classification, gender, age, residential area size, and the head of household’s education level. In this study, we utilized the health questionnaire and health examination data collected from the KNHANES samples. To analyze these data, we applied weights following the guidelines for complex sampling analysis provided by the Korea Centers for Disease Control and Prevention.

All statistical analyses used in the present study were conducted by complex sample data analysis based on SPSS 21 for Windows (SPSS, Inc., Chicago, IL, USA). Continuous variables are presented as means ± standard errors (SEs), while categorical variables are expressed as percentages with corresponding SEs. In the comparison of sex differences in basic characteristics and lifestyle characteristics, Rao-Scott chi-squared tests were conducted for categorical variables, and t tests based on a general linear model were performed for continuous variables. In complex sample analysis, the general linear model is utilized for various statistical analyses, including t tests. The general linear model allows for the incorporation of complex sampling designs, such as stratification and clustering, by using appropriate weights. Therefore, in our study, which involved complex sample analysis, we performed t tests using the general linear model instead of simple t tests. The demographic characteristics and variables of the subjects are presented in Table 1. Regarding the absolute and relative HGS indices and anthropometric indices, a binary logistic regression model was used to examine the association between arthritis and each index after standardization. We confirmed that there was no multicollinearity between the variables in the binary logistic regression model using the variance inflation factor, and we verified the linearity between the logit of the dependent variable and the independent variables through the Box-Tidwell test. Additionally, an effect size test of the large dataset was conducted using Cohen’s d and confirmed for all data sets according to the medium category of approximately 0.5. For the assumption that the observations are independent, we checked plots of residuals against samples and found random patterns of plots.

**Table 1:** Demographic characteristics of the subjects.

Variables		Men			Women			
	p value*	Nonarthritis	Arthritis	p value	Nonarthritis		Arthritis	p value
Number		6,668	699		6,584		2,909	
Age (years)	<0.001	60.84±0.14	65.86±0.40	<0.001	60.43±0.14		66.96±0.20	<0.001
Residential area	0.274			<0.001				<0.001
Urban		80.60 (1.30)	76.80 (2.20)		82.20 (1.20)		77.80 (1.60)	
Rural		19.40 (1.30)	23.20 (2.20)		17.80 (1.20)		22.20 (1.60)	
Marital status	<0.001			<0.001				<0.001
Married		90.81 (0.43)	89.35 (1.40)		74.60 (0.70)		60.90 (1.10)	
Single (widowed, divorced, etc.)		9.19 (0.43)	10.65 (1.40)		25.40 (0.70)		39.10 (1.10)	
Living situation	<0.001			<0.001				<0.001
With family		92.31 (0.38)	91.89 (1.07)		88.20 (0.50)		79.60 (0.90)	
Alone		7.69 (0.38)	8.11 (1.07)		11.80 (0.50)		20.40 (0.90)	
Education level	<0.001			<0.001				<0.001
< = Elementary school		19.00 (0.60)	37.17 (2.11)		31.90 (0.80)		58.20 (1.10)	
Middle school		15.60 (0.60)	18.34 (1.73)		16.20 (0.60)		16.20 (0.80)	
High school		33.30 (0.70)	27.89 (2.07)		34.10 (0.80)		18.60 (0.90)	
> = University		32.20 (0.90)	16.59 (1.79)		17.70 (0.70)		6.90 (0.50)	
Occupation	<0.001			<0.001				<0.001
White-collar worker		13.09 (0.56)	6.16 (1.21)		6.00 (0.40)		1.60 (0.30)	
Office worker		9.76 (0.50)	4.13 (0.89)		4.90 (0.30)		1.30 (0.20)	
Service worker		9.32 (0.48)	7.86 (1.30)		18.20 (0.60)		12.40 (0.70)	
Farmer or fisher		7.27 (0.55)	10.31 (1.35)		3.30 (0.30)		5.60 (0.60)	
Blue-collar worker		24.24 (0.71)	18.90 (2.01)		3.40 (0.30)		2.40 (0.40)	
Elementary occupations		9.50 (0.43)	9.56 (1.24)		13.60 (0.50)		14.10 (0.80)	
Unemployed (housewife, etc.)		26.81 (0.66)	43.08 (2.17)		50.60 (0.80)		62.50 (1.10)	
Household income	<0.001			<0.001				<0.001
Low		16.99 (0.57)	33.63 (2.02)		21.54 (0.68)		39.00 (1.10)	
Middle-low		24.69 (0.67)	27.39 (1.98)		25.29 (0.67)		25.40 (0.90)	
Middle-high		25.65 (0.69)	20.29 (1.86)		24.01 (0.67)		20.10 (0.90)	
High		32.68 (0.86)	18.69 (1.89)		29.15 (0.83)		15.60 (0.90)	
Stress	<0.001			<0.001				<0.001
Extreme		3.01 (0.24)	2.70 (0.70)		4.10 (0.30)		6.30 (0.50)	
High		15.45 (0.54)	14.20 (1.50)		17.40 (0.50)		20.90 (0.90)	
Slight		59.46 (0.69)	57.90 (2.40)		57.90 (0.70)		51.2 (1.10)	
Rare		22.08 (0.54)	25.10 (2.00)		20.60 (0.60)		21.5 (0.90)	
Alcohol consumption	<0.001			<0.001				<0.001
Never drinker		5.50 (0.30)	6.30 (1.00)		21.69 (0.61)		29.50 (1.00)	
Former drinker 1 year prior		15.00 (0.50)	19.70 (1.70)		21.53 (0.58)		23.60 (0.90)	
<1 per month		10.10 (0.40)	9.90 (1.30)		23.57 (0.60)		22.90 (0.90)	
1 per month		7.90 (0.40)	7.70 (1.10)		10.36 (0.45)		8.20 (0.60)	
2~4 per month		22.70 (0.60)	19.90 (1.90)		14.31 (0.53)		9.80 (0.60)	
2~3 per week		22.80 (0.60)	20.20 (1.90)		6.24 (0.37)		4.30 (0.40)	
> = 4 per week		15.90 (0.50)	16.30 (1.80)		2.30 (0.21)		1.70 (0.30)	
Smoking status	<0.001			<0.001				<0.001
Everyday		27.60 (0.70)	21.60 (2.00)		2.55 (0.24)		2.50 (0.40)	
Sometimes		3.30 (0.30)	1.80 (0.60)		0.96 (0.15)		0.50 (0.10)	
Past		50.90 (0.70)	60.50 (2.20)		3.08 (0.24)		3.50 (0.40)	
Never		18.30 (0.60)	16.10 (1.50)		93.42 (0.37)		93.50 (0.60)	
Walking exercise per week (min)	<0.001	276.20±6.12	253.59±14.97	0.152	255.15±5.23		217.03±7.07	<0.001
Sleep duration (hours)	<0.001	6.85±0.02	6.91±0.06	0.372	6.75±0.02		6.61±0.03	<0.001
Menopause	-			-				<0.001
No		-	-		26.08 (0.68)		15.30 (0.70)	
Yes		-	-		73.92 (0.68)		84.70 (0.70)	
Blood pressure								
SBP (mmHg)	0.388	123.59±0.24	125.41±0.66	0.010	122.25±0.27		126.65±0.38	<0.001
DBP (mmHg)	<0.001	77.89±0.16	75.38±0.45	<0.001	75.31±0.14		74.40±0.23	<0.001
Anthropometrics								
Height (cm)	<0.001	168.32±0.09	166.65±0.28	<0.001	155.48±0.09		153.54±0.13	<0.001
Weight (kg)	<0.001	68.77±0.15	68.41±0.41	0.411	57.56±0.12		59.06±0.20	<0.001
Body mass index (kg/m2)	0.049	24.23±0.04	24.60±0.13	0.006	23.80±0.05		25.02±0.07	<0.001
Waist circumference (cm)	<0.001	86.89±0.12	88.92±0.38	<0.001	80.93±0.15		84.96±0.21	<0.001
Waist-to-height ratio	<0.001	0.52±0.00	0.53±0.00	<0.001	0.52±0.00		0.55±0.00	<0.001
Dominant hand	0.013			0.017				0.018
Right		87.46 (0.51)	85.80 (1.60)		89.00 (0.50)		88.80 (0.70)	
Left		5.63 (0.34)	4.80 (0.90)		4.50 (0.30)		5.00 (0.40)	
Both		6.91 (0.39)	9.40 (1.30)		6.50 (0.40)		6.20 (0.60)	
Absolute HGS (kg)	<0.001	36.34±0.12	32.90±0.36	<0.001	21.61±0.08		19.71±0.11	<0.001
Relative HGS								
HGS-HT (kg/height)	<0.001	0.22±0.00	0.20±0.00	<0.001	0.14±0.00		0.13±0.00	<0.001
HGS-WT (kg/weight)	<0.001	0.53±0.00	0.48±0.00	<0.001	0.38±0.00		0.34±0.00	<0.001
HGS-BMI (kg/BMI)	<0.001	1.51±0.01	1.35±0.02	<0.001	0.92±0.00		0.80±0.00	<0.001
HGS-WC (kg/WC)	<0.001	0.42±0.00	0.37±0.00	<0.001	0.27±0.00		0.23±0.00	<0.001
HGS-WHtR (kg/WHtR)	<0.001	71.02±0.26	62.30±0.76	<0.001	42.19±0.20		36.14±0.23	<0.001

SBP: systolic blood pressure, DBP: diastolic blood pressure, HGS: hand grip strength, HT: height, WT: weight, BMI: body mass index, WC: waist circumference, WHtR: waist-to-height ratio.

Continuous data are represented as the means ± SEs (standard errors). Categorical data are represented as percentages (SEs).

P value* indicates the p values for sex differences between all men and women. The p values were obtained from Rao-Scott chi-squared tests for categorical variables and from a general linear model for continuous variables between the arthritis group and the nonarthritis group.

Three models were built based on different covariates: the crude model, which involved no adjustments; Model 1, which included adjustments for age and BMI; and Model 2, which included adjustments for residential area, education level, occupation, household income, alcohol consumption, smoking status, walking exercise, age, and BMI. These models were constructed using the binary logistic regression model. ORs are presented with 95% confidence intervals with p values (a significance level of 0.05) for each model. The adjustment variables in models 1 and 2 were selected based on previous studies on HGS measurement and arthritis. Common confounding variables in medicine are the attributes of subjects, and most studies related to diseases consider confounders based on sociodemographic and socioeconomic characteristics. Age, BMI, exercise, and occupation have been used in previous studies [2, 11, 13, 23, 24, 27, 38] and were selected as adjustment variables because these factors may influence results related to HGS or arthritis. Additionally, we adjusted for the potential confounders of education level, alcohol consumption, smoking status, and household income associated with HGS or arthritis based on previous studies [2, 13, 38]. We added one potential confounder of residential area related to regional differences, income, and environment.

## Results

### Demographic characteristics of the subjects

Table 1 shows the demographic characteristics of the nonarthritis and arthritis groups. A total of 16,860 subjects aged ≥ 50 years (men = 7,367, women = 9,493) were included in the analysis. The final analysis dataset consisted of 13,252 subjects (men = 6,668, women = 6,584) without arthritis and 3,608 subjects (men = 699, women = 2,909) with arthritis. The overall prevalence of arthritis in the study was 21.40%. Specifically, the prevalence of arthritis was 9.49% among men and 30.64% among women; the prevalence of arthritis among women was approximately three times higher than the prevalence among men.

We analyzed statistically significant differences between men and women and between the arthritis group and the nonarthritis group. All indices except for residential area and systolic blood pressure (SBP) showed significant differences between men and women. In addition, among men, all indices except for walking exercise, sleep duration, and weight differed significantly between the nonarthritis and arthritis groups, whereas among women, all indices differed significantly between the two groups. The average age of the arthritis group was older than that of the nonarthritis group. Both men and women in the arthritis group were likely to be single and live alone and had a lower educational level and income and a higher unemployment rate than people in the nonarthritis group. Stress showed a clear difference between men and women, with women in the arthritis group tending to be more stressed than men. The rates of smoking and drinking were slightly lower in the arthritis group than in the nonarthritis group among both men and women. The walking exercise time tended to be lower in the arthritis group than in the nonarthritis group among both men and women, but it was not statistically significant among men. In addition, women in the arthritis group were likely to sleep less and have a higher menopause rate than women in the nonarthritis group.

With respect to blood pressure and anthropometric indices, among both men and women, the arthritis group tended to have slightly higher SBP, slightly lower diastolic blood pressure (DBP), shorter height, heavier weight, and greater waist circumference (WC) and waist-to-height ratio (WHtR) than the nonarthritis group. Body mass index (BMI) was similar in the two groups for men, whereas the BMI of women in the arthritis group was higher than the other groups. Among men, the proportion of ambidexterity in the arthritis group was higher than the other groups, while the percentage of women who were ambidextrous in the arthritis group was slightly lower. Both men and women in the arthritis group tended to have lower HGS than men and women in the nonarthritis group.

### Associations of arthritis with anthropometry and HGS

Tables 2 and 3 show the associations of arthritis with anthropometric and HGS indices. Model 1 was adjusted for age and BMI, and Model 2 was adjusted for residential area, education level, occupation, household income, alcohol consumption, smoking status, walking exercise, age, and BMI. In the crude analysis, all anthropometric and HGS indices except for weight were associated with arthritis in men, and all indices were associated with arthritis in women. In particular, among all the indices, age showed a more strongly positive association with arthritis in both groups of men. In Models 1 and 2 adjusted for various covariates, all HGS indices related to anthropometry were associated with arthritis in both men and women, whereas simple anthropometric indices showed differences between men and women. For men, among the anthropometric indices, only WC and WHtR were associated with arthritis in Model 1 and Model 2. In particular, WHtR showed a more significant positive association with arthritis than the other indices. The absolute HGS and all relative HGS indices (HGS-HT, HGS-WT, HGS-BMI, HGS-WC, and HGS-WHtR) showed a negative association with arthritis in men. The magnitudes of association between the absolute HGS index and arthritis were similar to the associations between the relative HGS indices and the disease. Among women, none of the anthropometric indices showed a significant association with arthritis in Models 1 and 2, unlike men. However, all absolute and relative HGS indices showed a significant association with arthritis similar to men. The magnitudes of associations between the absolute HGS index and arthritis and between relative HGS indices and arthritis were similar. Additionally, the magnitude of the association between all HGS indices and arthritis was higher for men than for women in Models 1 and 2.

**Table 2:** Associations of arthritis with anthropometric indices and absolute and relative HGS indices among men.

Variables	Crude		Model 1		Model 2	
	OR (95% CI)	p value	Adj. OR (95% CI)	Adj. p value	Adj. OR (95% CI)	Adj. p value
Age	1.72 (1.57–1.88)	<0.001				
Anthropometrics						
Height	0.76 (0.69–0.83)	<0.001	0.91 (0.82–1.01)	0.071	0.97 (0.87–1.08)	0.541
Weight	0.96 (0.88–1.05)	0.413	0.82 (0.66–1.00)	0.052	0.92 (0.75–1.13)	0.425
Body mass index	1.14 (1.04–1.24)	0.005				
Waist circumference	1.28 (1.17–1.41)	<0.001	1.25 (1.04–1.52)	0.019	1.26 (1.04–1.53)	0.017
Waist-to-height ratio	1.43 (1.30–1.57)	<0.001	1.48 (1.21–1.82)	<0.001	1.37 (1.12–1.68)	0.003
Absolute HGS (kg)	0.64 (0.58–0.70)	<0.001	0.78 (0.69–0.88)	<0.001	0.81 (0.72–0.92)	0.001
Relative HGS						
HGS-HT	0.65 (0.60–0.72)	<0.001	0.79 (0.71–0.89)	<0.001	0.82 (0.73–0.92)	<0.001
HGS-WT	0.64 (0.58–0.70)	<0.001	0.81 (0.72–0.90)	<0.001	0.82 (0.73–0.93)	0.001
HGS-BMI	0.60 (0.55–0.66)	<0.001	0.77 (0.68–0.87)	<0.001	0.81 (0.71–0.92)	0.001
HGS-WC	0.59 (0.54–0.65)	<0.001	0.76 (0.67–0.85)	<0.001	0.79 (0.70–0.89)	<0.001
HGS-WHtR	0.58 (0.53–0.64)	<0.001	0.75 (0.66–0.85)	<0.001	0.79 (0.69–0.89)	<0.001

HGS: handgrip strength, HT: height, WT: weight, BMI: body mass index, WC: waist circumference, WHtR: waist-to-height ratio, OR: odds ratio.

OR and p values were obtained from the crude and adjusted analyses using complex sample binary logistic regression. Odds ratios were estimated with 95% confidence intervals.

Model 1 adjusted for age and BMI.

Model 2 adjusted for residential area, education level, occupation, household income, alcohol consumption, smoking status, walking exercise, age, and BMI.

**Table 3:** Associations of arthritis with anthropometric indices and absolute and relative HGS indices among women.

Variables	Crude		Model 1		Model 2	
	OR (95% CI)	p value	Adj. OR (95% CI)	Adj. p value	Adj. OR (95% CI)	Adj. p value
Age	2.00 (1.90–2.11)	<0.001				
Anthropometrics						
Height	0.72 (0.68–0.76)	<0.001	1.02 (0.96–1.09)	0.481	1.07 (1.00–1.14)	0.056
Weight	1.18 (1.13–1.25)	<0.001	1.05 (0.93–1.18)	0.457	1.13 (1.00–1.28)	0.056
Body mass index	1.44 (1.37–1.52)	<0.001				
Waist circumference	1.56 (1.48–1.65)	<0.001	1.07 (0.96–1.19)	0.239	1.04 (0.94–1.16)	0.432
Waist-to-height ratio	1.70 (1.61–1.80)	<0.001	1.03 (0.92–1.16)	0.620	0.96 (0.85–1.08)	0.534
Absolute HGS (kg)	0.69 (0.65–0.72)	<0.001	0.91 (0.86–0.97)	0.003	0.92 (0.87–0.98)	0.008
Relative HGS						
HGS-HT	0.71 (0.68–0.75)	<0.001	0.92 (0.86–0.97)	0.002	0.92 (0.87–0.97)	0.004
HGS-WT	0.62 (0.59–0.66)	<0.001	0.89 (0.84–0.95)	<0.001	0.89 (0.84–0.95)	<0.001
HGS-BMI	0.58 (0.55–0.62)	<0.001	0.89 (0.83–0.95)	<0.001	0.90 (0.84–0.96)	0.002
HGS-WC	0.59 (0.56–0.62)	<0.001	0.89 (0.83–0.95)	<0.001	0.90 (0.84–0.96)	0.002
HGS-WHtR	0.58 (0.55–0.61)	<0.001	0.89 (0.83–0.95)	<0.001	0.91 (0.85–0.97)	0.004

HGS: handgrip strength, HT: height, WT: weight, BMI: body mass index, WC: waist circumference, WHtR: waist-to-height ratio, OR: odds ratio.

OR and p values were obtained from the crude and adjusted analyses using complex sample binary logistic regression. Odds ratios were estimated with 95% confidence intervals.

Model 1 adjusted for age and BMI.

Model 2 adjusted for residential area, education level, occupation, household income, alcohol consumption, smoking status, walking exercise, age, and BMI.

## Discussion

HGS is a predictor of cardiovascular-related mortality [1–4], all-cause mortality and cardiovascular diseases [3, 4]. In this study, we examined the association of the absolute and relative HGS indices with arthritis in a Korean population. All HGS indices were highly associated with arthritis in both men and women. Subjects with arthritis had lower HGS values than subjects without arthritis. Additionally, the magnitudes of associations of arthritis with the absolute HGS index and relative HGS indices were similar, and the magnitude of association between all HGS indices and arthritis was higher for men than for women in Models 1 and 2.

In the association between absolute HGS and arthritis, several studies have reported that HGS is significantly related to arthritis and is a useful predictor of functional disability due to arthritis [10–15, 20–22]. It was suggested that arthritis patients had lower HGS values than normal subjects through comparison of HGS and pinch strength between a rheumatoid arthritis group and a control group [10]. The study emphasized that the reduction in HGS and pinch strength was an important predictor of functional disability in arthritis patients. Žura et al. [11] investigated differences in HGS between 100 men and women with rheumatoid arthritis compared with 100 healthy subjects and reported that both men and women with rheumatoid arthritis showed statistically weaker HGS than healthy subjects and that the magnitude of the association was greater for men than for women. Chen et al. [12] tested the relationship of HGS with health status among patients with rheumatoid arthritis and suggested that patients with arthritis had lower HGS and more functional limitations than subjects without arthritis. Lee et al. [13] examined the association of HGS with rheumatoid arthritis and diabetes among older Korean adults and argued that stronger HGS was related to a lower prevalence of rheumatoid arthritis and diabetes. They derived their results by focusing on data on older adults and rheumatoid arthritis based on the same data used in this study. Additionally, Da Rosa Iop et al. [15] investigated the capacity for maximum HGS between women with rheumatoid arthritis and healthy women. They found that women with rheumatoid arthritis showed a decreased ability to achieve maximum HGS compared to healthy women irrespective of dominance. Amorim et al. [22] examined the association between anthropometric indices, including HGS, and low or high functional performance in terms of several chronic diseases in older adults. They reported that functional performance was significantly associated with arthritis, and the high-functioning group had lower WHtR and higher HGS than the low-functioning group. Our findings are consistent with the results of previous studies [11–13, 15] that indicated that subjects with arthritis had lower HGS values than normal subjects and that higher HGS contributed to a decreased prevalence of arthritis. Additionally, our findings are in accordance with the finding that the magnitude of the association between HGS and arthritis was higher for men than for women [11].

Regarding relative HGS indices (calculated by dividing by BMI or body weight), studies on the relationship between relative HGS and arthritis are very difficult to find in the literature. However, recent studies have reported associations between relative HGS indices and various chronic diseases, such as cardiometabolic diseases, musculoskeletal diseases, liver enzyme concentrations, fatty liver disease, and cancers [38–43]. For example, Ahn et al. [38] tested the association between relative HGS and osteoporosis in an older Korean population. They used relative HGS as the maximum HGS divided by BMI, as suggested by Choquette et al. [39], and reported that relative HGS was related to osteoporosis in the left hand in women. Parra-Soto et al. [40] tested the associations of relative and absolute HGS with several cancers based on UK Biobank data. They calculated relative HGS indices divided by height, weight, BMI, and body fat mass and reported that head, neck, and breast cancers could be predicted by relative HGS. Additionally, Kim et al. [41] examined the association of musculoskeletal diseases and metabolic abnormalities with relative HGS (calculated as maximal HGS divided by BMI) in older subjects and found that relative HGS was associated with risk factors for diabetes, hypertension, cerebrovascular accidents, and osteoarthritis among both men and women. These studies argued that relative HGS indices may be a useful indicator of several chronic diseases.

The exact mechanism of association between arthritis and HGS or muscular strength remains uncertain [44]. There are several possible explanations based on biological and pathophysiological mechanisms. Arthritis patients show reduced HGS [10, 45–48], and HGS is a key indicator of muscle strength and arthritis status [17]. Rheumatoid arthritis is a chronic systemic inflammatory joint disease that causes reduced HGS and muscle strength [10, 45–48]. Generally, the reduction in HGS and skeletal muscle function is induced by various factors in the elderly population, such as aging, hormonal imbalances, chronic inflammation, insulin resistance, oxidative stress, poor nutrients and obesity [45, 49–53]. In inflammation, inflammatory cytokine interleukin-6 (IL) levels and tumor necrosis factor-receptor 1 (TNF-r1) are higher in the elderly population than in young and middle-aged adults [49, 51, 52, 54]. The increase in interleukin-6 levels is related to large reductions in muscle strength [49, 51, 54], and TNF-r1 is a risk factor for reduced muscle strength [49, 52]. Additionally, increased C-reactive protein (CRP) leads to low HGS and reduced physical performance [54–56]. CRP, which is produced in the liver, increases when inflammation occurs [56]. These inflammatory markers are related to decreased muscle mass in the elderly population, and muscle mass itself may act as a regulator or modulator of inflammatory markers [56]. On the other hand, aging is associated with a reduction in the ability to maintain glucose levels due to decreased sensitivity to insulin [49]. In older subjects, insulin resistance and type 2 diabetes are associated with an excessive reduction in muscle mass and are related to greater loss of skeletal muscle compared to nondiabetic counterparts independent of age, ethnicity, obesity, and sex [49, 53]. These biological and pathological risk factors are associated with low muscle strength and HGS and lead to arthritis.

Although many studies have suggested the usefulness of HGS as an indicator of several diseases [57–61], the reliability and validity of HGS are not yet sufficient due to the lack of standard protocols, conditions, and consensus on HGS measurement [16, 57, 58, 60]. A protocol for HGS measurement should consider many factors, such as maximum or mean HGS, dynamometer equipment, posture, hand dominance and size, pretest or warm-up test, grip duration, test intervals, and the number of tests [57, 58]. Recently, some studies have introduced the reliability of HGS measurements and posture and have presented consensus on the use of protocols in clinical settings. Mehmet et al. [57] and Roberts et al. [58] reported that a Jamar hand dynamometer was most widely used and that there was variation in HGS according to postures or protocols. Bobos et al. [59] compared HGS measurement protocols in a systematic review and meta-analysis. They found a remarkable intraclass correlation coefficient of 0.92 for the reliability estimates of HGS testing in healthy subjects in eight studies and minimum clinically important difference scores for HGS of 5.0 kg and 6.2 (dominant and nondominant, respectively) in stroke patients. Watanabe et al. [60] investigated the reliability of HGS and the effects of posture in 100 healthy hospital workers and reported that there was no difference in the minimum HGS between sitting and standing postures. Studies have suggested fundamental guidelines or protocols for HGS measurements, such as the American Society of Hand Therapists (ASHT) and Southampton, but protocols or guidelines for HGS measurement in specific diseases should consider more factors, such as hand size, wrist, elbow, shoulder, posture, height, weight, and age [57, 58, 61]. For example, reference equations of normative HGS in the United States include age, sex, dominance, height, and weight [61], and HGS is significantly correlated with height, weight, age, and WC [61, 62]. However, comparisons of the absolute HGS measurements and the relative HGS measurements combined with anthropometry in patients with arthritis have not yet been conducted. Our results may contribute to the design of fundamental guidelines for HGS measurement in arthritis because we report the comparison between the relative HGS indices combined with anthropometry and the absolute HGS index in arthritis.

The present study has several limitations. First, we did not establish a cause-and-effect relationship due to the cross-sectional design of this study. Second, this study focused on arthritis, including both osteoarthritis and rheumatoid arthritis, in an older Korean population. However, arthritis is mainly classified as osteoarthritis or rheumatoid arthritis, so further study is needed to reveal differences between the two conditions in terms of various HGS indices or anthropometric indices. Finally, respondent data on arthritis diagnosis based on face-to-face health interviews may lead to recall bias. To avoid recall bias, interviews were conducted with experts or well-trained staff rather than by self-administered questionnaires. Despite the limitations, the present study has strengths; for example, the results of this study are statistically powerful because data from the Korea National Health and Nutrition Survey (KNHANES) support a nationally representative sample of the Korean population. To our knowledge, the present study is the first report to compare the association of absolute and relative HGS indices with arthritis. It suggests for the first time the use of relative indices such as HGS-WHtR and HGS-WC (calculated by dividing the WHtR and WC) in the protocols of HGS measurements.

## Supporting information

S1 Checklist STROBE statement—checklist of items that should be included in reports of observational studies. (DOCX)
