# Supplementary material for: Association between arthritis and hand grip strength indices combined with anthropometry in an older Korean population
Source: PLoS One. 2023 Aug 31;18(8):e0291046. doi: 10.1371/journal.pone.0291046 (PMC10470972; doi:10.1371/journal.pone.0291046)
Supplement: S1 Checklist — (DOCX) [file pone.0291046.s001.docx]

STROBE Statement—checklist of items that should be included in reports of observational studies

|  | Item No. | Recommendation | Page  No. | Relevant text from manuscript |
| --- | --- | --- | --- | --- |
| **Title and abstract** | 1 | (*a*) Indicate the study’s design with a commonly used term in the title or the abstract | 2 | In a large-scale cross-sectional study … |
|  |  | (*b*) Provide in the abstract an informative and balanced summary of what was done and what was found | 2 | refer to the Abstract section. |
| Introduction | | | |  |
| Background/rationale | 2 | Explain the scientific background and rationale for the investigation being reported | 4-5 | However, these studies did not consider relative HGS indices, … |
| Objectives | 3 | State specific objectives, including any prespecified hypotheses | 5 | The objectives of this study were to examine the association between arthritis and HGS indices … |
| Methods | | | |  |
| Study design | 4 | Present key elements of study design early in the paper | 5 | In this large-scale cross-sectional study, we used the KNHANES dataset from 2014 to 2019, … |
| Setting | 5 | Describe the setting, locations, and relevant dates, including periods of recruitment, exposure, follow-up, and data collection | 5-6 | The Korean National Health and Nutrition Examination Survey (KNHANES) is … |
| Participants | 6 | (*a*) *Cohort study*—Give the eligibility criteria, and the sources and methods of selection of participants. Describe methods of follow-up  *Case-control study*—Give the eligibility criteria, and the sources and methods of case ascertainment and control selection. Give the rationale for the choice of cases and controls  *Cross-sectional study*—Give the eligibility criteria, and the sources and methods of selection of participants | 6-7 and Figure 1 | A total of 47,309 (men = 21,566, women = 25,743) subjects participated in the health interview survey … |
|  |  | (*b*) *Cohort study*—For matched studies, give matching criteria and number of exposed and unexposed  *Case-control study*—For matched studies, give matching criteria and the number of controls per case | N/A | N/A |
| Variables | 7 | Clearly define all outcomes, exposures, predictors, potential confounders, and effect modifiers. Give diagnostic criteria, if applicable | 6-9 | refer to the Definition of arthritis, Measurement, and Statistical analysis subsections. |
| Data sources/ measurement | 8* | For each variable of interest, give sources of data and details of methods of assessment (measurement). Describe comparability of assessment methods if there is more than one group | *5-9* | refer to the Study population and data sources, Definition of arthritis, Measurement, and Statistical analysis subsections. |
| Bias | 9 | Describe any efforts to address potential sources of bias | 7 | To overcome respondent recall bias regarding the diagnosis of arthritis, … |
| Study size | 10 | Explain how the study size was arrived at | 6 and Figure 1 | Data on arthritis were collected through face-to-face health interviews with well-trained staff and determined by responses to two questions. |

Continued on next page

| Quantitative variables | 11 | Explain how quantitative variables were handled in the analyses. If applicable, describe which groupings were chosen and why | 6 and Figure 1 | Subjects who answered “yes” to at least one of the two questions were placed in the arthritis group, … |
| --- | --- | --- | --- | --- |
| Statistical methods | 12 | (*a*) Describe all statistical methods, including those used to control for confounding | 8-9 | Three models were built according to covariates: the crude analysis was a crude model; … |
|  |  | (*b*) Describe any methods used to examine subgroups and interactions | 8 | … a binary logistic regression model was used to examine the association between arthritis … |
|  |  | (*c*) Explain how missing data were addressed | Figure 1 | Refer to Figure 1 |
|  |  | (*d*) *Cohort study*—If applicable, explain how loss to follow-up was addressed  *Case-control study*—If applicable, explain how matching of cases and controls was addressed  *Cross-sectional study*—If applicable, describe analytical methods taking account of sampling strategy | 8 and Figure 1 | Therefore, complex sampling of clustering and weights with stratification related to … |
|  |  | (*e*) Describe any sensitivity analyses | N/A | N/A |
| Results | | | | |
| Participants | 13* | (a) Report numbers of individuals at each stage of study—eg numbers potentially eligible, examined for eligibility, confirmed eligible, included in the study, completing follow-up, and analysed | 11 | Table 1 shows the demographic characteristics of the nonarthritis and arthritis groups. A total of 16,680 subjects aged ≥ 50 years (men = 7,367, women = 9,493) … |
|  |  | (b) Give reasons for non-participation at each stage | 11 | The final analysis dataset consisted of 13,252 subjects (men = 6,668, women = 6,584) without arthritis … |
|  |  | (c) Consider use of a flow diagram | Figure 1 | refer to the Figure 1. |
| Descriptive data | 14* | (a) Give characteristics of study participants (eg demographic, clinical, social) and information on exposures and potential confounders | 11 | The overall prevalence of arthritis was 21.40%, 9.49% for men and 30.64% for women … |
|  |  | (b) Indicate number of participants with missing data for each variable of interest | Figure 1 | refer to the Figure 1. |
|  |  | (c) *Cohort study*—Summarise follow-up time (eg, average and total amount) | N/A | N/A |
| Outcome data | 15* | *Cohort study*—Report numbers of outcome events or summary measures over time | N/A | N/A |
|  |  | *Case-control study—*Report numbers in each exposure category, or summary measures of exposure | N/A | N/A |
|  |  | *Cross-sectional study—*Report numbers of outcome events or summary measures | *13-14* | Refer to the Tables 2 and 3 |
| Main results | 16 | (*a*) Give unadjusted estimates and, if applicable, confounder-adjusted estimates and their precision (eg, 95% confidence interval). Make clear which confounders were adjusted for and why they were included | *13-14* | Refer to the Tables 2 and 3 |
|  |  | (*b*) Report category boundaries when continuous variables were categorized | N/A | N/A |
|  |  | (*c*) If relevant, consider translating estimates of relative risk into absolute risk for a meaningful time period | N/A | N/A |

Continued on next page

| Other analyses | 17 | Report other analyses done—eg analyses of subgroups and interactions, and sensitivity analyses |  | N/A |
| --- | --- | --- | --- | --- |
| Discussion | | | | |
| Key results | 18 | Summarise key results with reference to study objectives | 15 | All HGS indices were highly associated with arthritis in both men and women. Subjects with arthritis had lower HGS values than normal subjects. |
| Limitations | 19 | Discuss limitations of the study, taking into account sources of potential bias or imprecision. Discuss both direction and magnitude of any potential bias | 19 | The present study has several limitations. First, we did not … |
| Interpretation | 20 | Give a cautious overall interpretation of results considering objectives, limitations, multiplicity of analyses, results from similar studies, and other relevant evidence | 18 | The exact mechanism of association between arthritis and HGS or muscular strength … |
| Generalisability | 21 | Discuss the generalisability (external validity) of the study results | 20 | … the present study is the first report comparing the association of absolute and relative HGS indices with arthritis and suggests, for the first time, using relative HGS indices such as the HGS-WHtR and HGS-WC … |
| Other information | |  | | |
| Funding | 22 | Give the source of funding and the role of the funders for the present study and, if applicable, for the original study on which the present article is based | - | We submitted the source of funding and the role of the funders in step of submission stage (site). |

*Give information separately for cases and controls in case-control studies and, if applicable, for exposed and unexposed groups in cohort and cross-sectional studies.

**Note:** An Explanation and Elaboration article discusses each checklist item and gives methodological background and published examples of transparent reporting. The STROBE checklist is best used in conjunction with this article (freely available on the Web sites of PLoS Medicine at http://www.plosmedicine.org/, Annals of Internal Medicine at http://www.annals.org/, and Epidemiology at http://www.epidem.com/). Information on the STROBE Initiative is available at www.strobe-statement.org.
